# Supplementary material for: Fecal microbiota profiles of growing pigs and their relation to growth performance
Source: PLoS One. 2024 May 6;19(5):e0302724. doi: 10.1371/journal.pone.0302724 (PMC11073740; doi:10.1371/journal.pone.0302724)
Supplement: S1 Fig — The most correlated genera are listed and their correlations with the axes are shown. A human keystone bacterium Christensenella minuta is studied for reducing obesity in humans. Akkermansia muciniphila is used to treat obesity and diabetes in humans. (DOCX) [file pone.0302724.s001.docx]

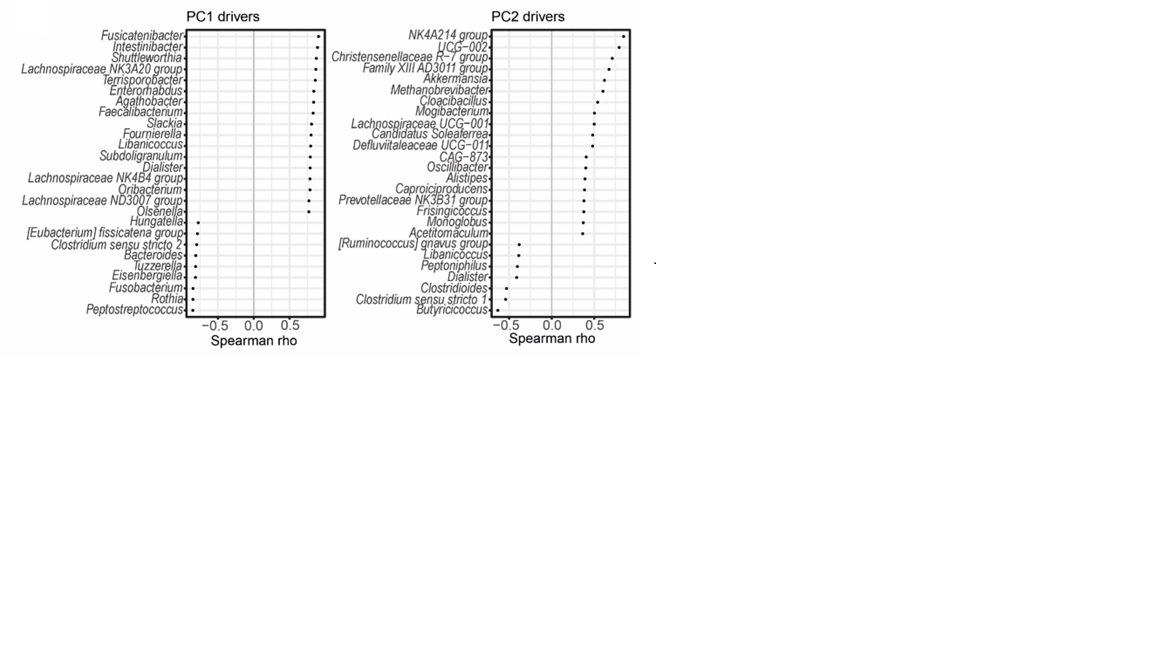


**Figure S1. Driver genera of principal component axes 1 (PC1) and 2 (PC2) of the Principal Coordinates Analysis in Figure 8 in Results.** The most correlated genera are listed and their correlations with the axes are shown. A human keystone bacterium Christensenella *minuta* is studied for reducing obesity in humans. *Akkermansia muciniphila* is used to treat obesity and diabetes in humans.
