# Supplementary material for: Fecal microbiota profiles of growing pigs and their relation to growth performance
Source: PLoS One. 2024 May 6;19(5):e0302724. doi: 10.1371/journal.pone.0302724 (PMC11073740; doi:10.1371/journal.pone.0302724)
Supplement: S2 Fig — Samples taken during the transitional and stable phases differ numerically from the initial exposure phase sample. (DOCX) [file pone.0302724.s002.docx]

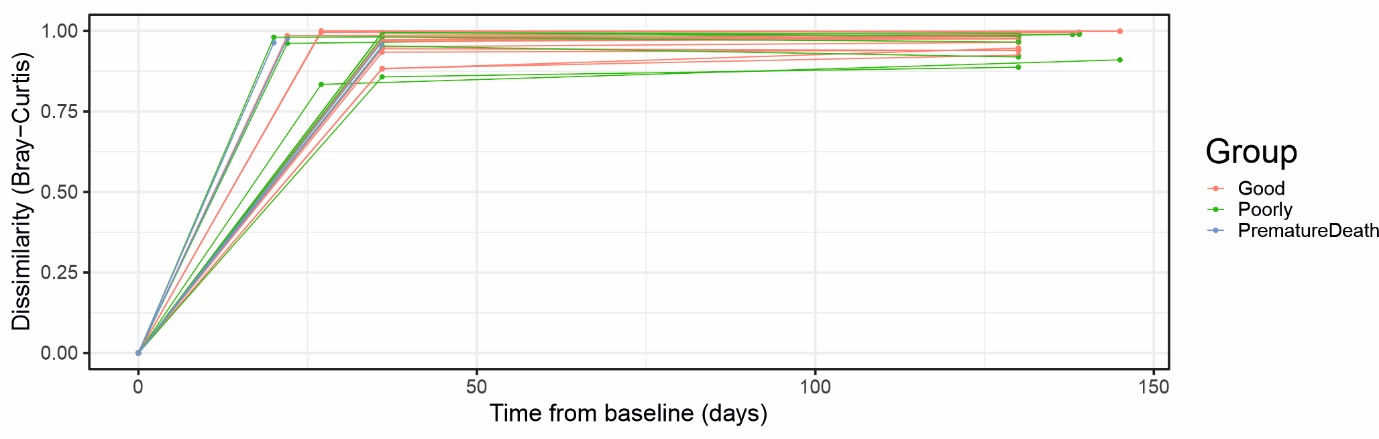


**Figure S2. Comparison of gut microbiome (dis)similarity of the development groups (Good n=13, Poorly n=8, and PrematureDeath n=4).** Samples taken during the transitional and stable phases differ numerically from the initial exposure phase sample.
