# Supplementary material for: Fecal microbiota profiles of growing pigs and their relation to growth performance
Source: PLoS One. 2024 May 6;19(5):e0302724. doi: 10.1371/journal.pone.0302724 (PMC11073740; doi:10.1371/journal.pone.0302724)
Supplement: S3 Fig — The a) alpha diversity and b) beta diversity analyses of dams of the pigs from the different development groups (Good, Poorly, and PrematureDeath) relating to Fig 10 in Results. (DOCX) [file pone.0302724.s003.docx]

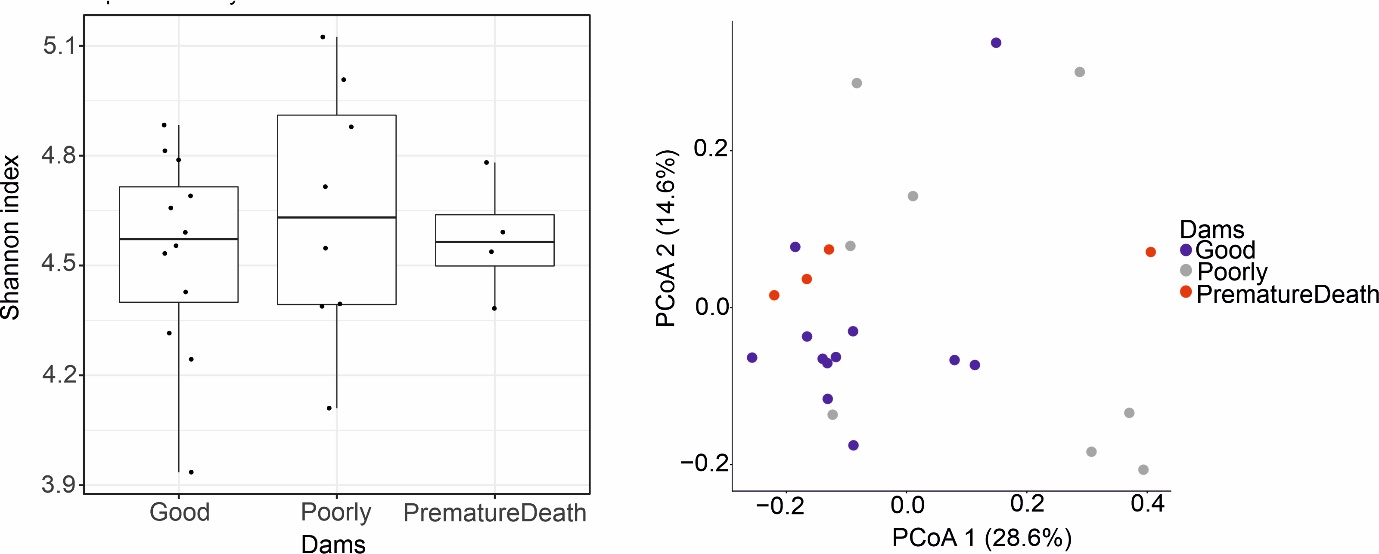


**Figure S3. Comparison of dam samples.** The a) alpha diversity and b) beta diversity analyses of dams of the pigs from the different development groups (Good, Poorly, and PrematureDeath) relating to Figure 10 in Results.
