# Supplementary material for: Fecal microbiota profiles of growing pigs and their relation to growth performance
Source: PLoS One. 2024 May 6;19(5):e0302724. doi: 10.1371/journal.pone.0302724 (PMC11073740; doi:10.1371/journal.pone.0302724)
Supplement: S1 Table — All parturitions on the farms were tracked for one week and all piglets born during the week were ear tagged for individual identification. (DOCX) [file pone.0302724.s004.docx]

**Table S1.** **Production information of sows giving birth during tracking week for all study farms.** All parturitions on the farms were tracked for one week and all piglets born during the week were ear tagged for individual identification.

| **Farm** | **Total number of sows** | **Number of sows giving birth during tracking week** | **Piglets born during tracking week** | **Live born piglets, average (range, SD)** | **Stillborn piglets,**  **average (range, SD)** | **Sow parity, average (range, SD)** |
| --- | --- | --- | --- | --- | --- | --- |
| Farm 1 | 1000 | 30 | 364 | 13.4  (4-22, 4.0) | 0.6  (0-3, 1.0) | 3.7  (1-8, 2.1) |
| Farm 2 | 1100 | 91 | 1461 | 18.8  (11-25, 3.2) | 2.8  (0-9, 2.0) | 2.9  (1-7, 1.7) |
| Farm 3 | 1271 | 39 | 541 | 15.0  (4-23, 3.8) | 1.0  (0.3, 1.0) | 2.6  (1-6, 1.4) |
